# Urinary Extracellular Vesicles in Chronic Kidney Disease: From Bench to Bedside?

**DOI:** 10.3390/diagnostics13030443

**Published:** 2023-01-26

**Authors:** Charlotte Delrue, Sander De Bruyne, Reinhart Speeckaert, Marijn M. Speeckaert

**Affiliations:** 1 Department of Nephrology, Ghent University Hospital, 9000 Ghent, Belgium; 2 Department of Diagnostic Sciences, Ghent University, 9000 Ghent, Belgium; 3 Department of Dermatology, Ghent University Hospital, 9000 Ghent, Belgium; 4 Research Foundation-Flanders (FWO), 1000 Brussels, Belgium

**Keywords:** urinary extracellular vesicles, chronic kidney disease, biomarkers

## Abstract

Extracellular vesicles are a diverse group of particles that include exosomes, microvesicles, and apoptotic bodies and are defined by size, composition, site of origin, and density. They incorporate various bioactive molecules from their cell of origin during formation, such as soluble proteins, membrane receptors, nucleic acids (mRNAs and miRNAs), and lipids, which can then be transferred to target cells. Extracellular vesicles/exosomes have been extensively studied as a critical factor in pathophysiological processes of human diseases. Urinary extracellular vesicles could be a promising liquid biopsy for determining the pattern and/or severity of kidney histologic injury. The signature of urinary extracellular vesicles may pave the way for noninvasive methods to supplement existing testing methods for diagnosing kidney diseases. We discuss the potential role of urinary extracellular vesicles in various chronic kidney diseases in this review, highlighting open questions and discussing the potential for future research.

## 1. Introduction

**Figure 1:**
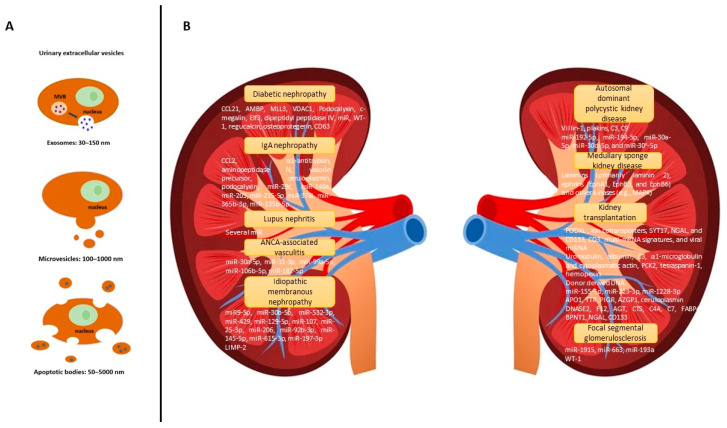
(**A**) The classification of extracellular urinary vesicles. Abbreviation: MVB: multivesicular bodies. (**B**) The potential value of urinary extracellular vesicles in chronic kidney diseases.

Extracellular vesicles (EVs) are particles that are naturally released from the cell and are separated by a lipid bilayer. EVs have piqued the scientific community’s interest due to their diverse roles in intercellular communication, pathogenesis, and drug and gene vector delivery and as potential biomarker reservoirs [1]. These vesicles can be released by a variety of cells, including immune system cells, platelets, and neurons, among others [2], and can be found in a variety of body fluids, including plasma, urine, and saliva [3,4]. The term EVs refers to various types of vesicles (exosomes, ectosomes (also called microvesicles), and apoptotic bodies), which differ in several ways (Figure 1A) [5,6]. Exosomes are 30–150 nm diameter vesicles derived from endosomal membrane inward budding, which results in the progressive accumulation of intraluminal vesicles within large multivesicular bodies. By fusing with the plasma membrane, these exosomes are released into the environment [6,7]. Ectosomes are larger than exosomes (100–1000 nm) and formed by direct budding of the plasma membrane [8]. Finally, dying cells expel membranous vesicles with variable shape and size known as apoptotic blebs [6].

The specific composition and function of EVs, which contain a subset of common proteins involved in biogenesis and trafficking, as well as a signature from the cell or tissue of origin, are explained by their different subcellular origin [6,9]. Because EV release and uptake are both dynamic processes regulated by a variety of mechanisms, it is difficult to accurately estimate the duration and concentration of EVs that a specific cell type would be exposed to in a physiological or pathophysiological scenario [10]. EVs modulate leukocyte adhesion, differentiation, and vascular function in inflammation, according to mechanistic data, and these studies have greatly improved our understanding of these pathophysiologic processes [11,12].

Exosomes can be extracted from a variety of body fluids, including blood, urine, saliva, seminal fluid, amniotic fluid, cerebrospinal fluid, bile, pericardial fluid, ascites, and pleural effusion [13]. Urine is a body waste fluid that is easy to obtain, making it an ideal fluid for biomarker determination and analysis. However, urine is a complex mixture of filtered and secreted proteins, salts, urea, and metabolites that can vary not only in physiological situations but also in chronic kidney diseases [14,15]. The protein composition of the urine is heavily influenced by the patient’s glomerular filtration rate (GFR), tubular metabolism, tubular reabsorption, diet, and hydration status, among other factors. Any tissue disorder or pathophysiological condition could cause a change in the concentration of a specific protein. Urinary EVs (uEVs) are thought to contain only about 3% of urine proteins. As a result, potential interesting biomarkers contained in EVs may simply go undetected due to dilution in whole urine [16,17]. Because of the strong correlation between kidney and uEV protein abundances, uEVs are an appealing noninvasive source of biomarkers for research into kidney physiology or disease [18]: for example, uEVs provide a noninvasive method for studying tubular transport in health and disease [19]. Thus, determining biomarkers specifically related to urinary EVs may be of interest in detecting changes in the kidney system [16,17].

Beginning with nephrogenesis, EVs appear to play an important role in kidney physiology. EVs derived from embryonic kidney stem cells modulate the secondary inductive interactions that control organogenesis between the ureteric bud and metanephric mesenchyme. They transport proteins that are important for extracellular matrix organization, cell shape, tissue integrity and homeostasis, and tissue morphogenesis [20]. Their cargo includes various miRNAs with regulatory properties that influence Wnt pathways, which trigger the nephrogenesis developmental program [20,21]. Ureteric bud exosomes interact with metanephric mesenchyme cells via various modes of exosomal material transfer. Exosomes as a whole enter the cell and release their RNA cargo into the cytoplasm. Furthermore, exosomes and their RNA cargo are transferred not only to the cytoplasm but also to the nucleus, which is critical in signal transduction and the exertion of exosome influence on the metabolism and phenotype of target cells. Exosomes appear to improve cellular organization and cell survival during the early stages of kidney development [20]. Intranephron communication can be mediated by uEVs. EVs, produced by tubular epithelial cells, are involved in the communication between the proximal and distal tubules [22]. They can also maintain contact with interstitial cells (fibroblasts and macrophages) and influence the immune response [23]. EVs are also released by glomerular mesangial cells and podocytes. EVs from podocytes can promote fibrotic signaling by communicating with proximal tubule epithelial cells [24]. The nephron mass influences the excretion of uEVs, and a nephrectomy reduces the uEV excretion less than expected based on nephron loss due to compensatory hypertrophy [25].

Since the first description of exosomes in urine in 2004 [26], urinary EVs have emerged as an appealing target for biomarker discovery in kidney disease. In the present review, we will discuss the potential value of uEVs in chronic kidney disease (CKD) and after kidney transplantation.

## 2. Chronic Kidney Disease

### 2.1. Diabetic Nephropathy

Diabetic nephropathy (DN) affects approximately 40% of people with diabetes mellitus (DM), the leading cause of CKD and end-stage kidney disease (ESKD) worldwide [27]. Despite the fact that estimated glomerular filtration rate (eGFR) and albuminuria are well-established biomarkers for DN, they are insufficient in patients who do not have sustained albuminuria or preserved eGFR [28]. There is an urgent need for accurate and dependable markers to detect early kidney dysfunction and structural DN lesions [29,30]. Furthermore, new biomarkers may provide a better understanding of the complex pathophysiological processes underlying DN [31].

#### 2.1.1. Animal Studies

A label-free liquid chromatography–tandem mass spectrometry (LC–MS/MS) approach was used to investigate the proteome changes in urinary exosomes isolated from Zucker diabetic fatty rats, a type 2 DN model. After prefiltration and tryptic digestion, exosomal proteins from urine samples of 20-week-old rats were pooled and analyzed, resulting in the identification and label-free quantification of 286 proteins. UniProt Knowledgebase (UniProtKB) revealed that the majority of identified proteins were membrane-associated or cytoplasmic and involved in transport, signaling, and cellular adhesion, all of which are typical functions of exosomal proteins [32].

In a rat model of early DN, regucalcin, also called senescence marker protein-30 (SMP30), was clearly detected in urinary exosomes from pooled healthy rat urine but not in diabetic rat urine exosomes, reflecting pathological tissue changes [33]. Regucalcin is a multifunctional protein that regulates cellular Ca^2+^ homeostasis, ascorbate biosynthesis, and oxidative stress [34,35]. Associative studies show that regucalcin is also involved in β-cell function, and regucalcin deficiency impairs insulin secretion [36] and contributes to the common deterioration of glucose tolerance in aging [37]. Endothelial cells with high glucose levels have lower regucalcin levels [38]. TNF-α and TGF-β1, which are present in the DN milieu, reduced regucalcin mRNA in cultured tubular cells [39]. Furthermore, there is evidence that regucalcin plays a role in kidney injury protection [40].

In another experimental diabetic mice model, miR-145 levels were twice as high in diabetic urinary exosomes as in nondiabetic animals [41]. TGF-β1 induced the expression of miR-145, a glomerular marker of mesangial cells [42,43]. miR-145 may be involved in high-glucose-induced mesangial cell hypertrophy as well as cytoskeleton remodeling [44]. Exosomes enriched in miR-145 were released by mesangial cells when exposed to high glucose levels, mirroring the overexpression observed in parent cells. This raises the intriguing possibility of urinary exosomes originating from mesangial cells with increased miR-145 content. High glucose levels may disrupt the complex process that leads to exosome formation/release from the multivesicular bodies [41].

#### 2.1.2. Human Studies

Tubular injury is an important component of the early course of DN that contributes to its development. Under DN conditions, active EV secretion may represent the tubule’s adaptive response to external stress. During the early stages of DN, endocytosis and EV release may be activated as interconnected pathways [45]. The transcriptome profile in urinary EVs may provide a noninvasive method for detecting histological changes in DN. CCL21 mRNA expression increased progressively with the progression of tubulointerstitial inflammation and was highest in the nodular sclerosis group (class III) of DN patients. CCL21 mRNA derived from urinary small EVs may serve as an early biomarker for identifying DN associated with pathogenesis. T-cell infiltration mediated by CCL21 mRNA may be a key mechanism of chronic inflammation in DN. CCL21 also demonstrated accurate diagnostic ability in distinguishing between incipient and overt DN. When DN patients were compared with DM patients and healthy controls, the number of small EVs secreted in urine increased, as did the expression of aquaporin 1 (AQP1, a marker of proximal tubules) and AQP2 (a marker of distal/collecting tubules) [46]. Elevated C-C motif chemokine ligand 21 (CCL21) mRNA from uEVs was more effective than eGFR and proteinuria in distinguishing early DN patients from DM. Moreover, CCL21 mRNA derived from small EVs correlated with proteinuria and eGFR [45]. Besides, the amount and activity of dipeptidyl peptidase IV (DPPIV/CD26) in urinary microvesicles derived from proximal tubule cells has been shown to correlate with the progression of DN in type 2 DM patients, implying an early tubular impairment, which may be considered an early marker of kidney damage even before the onset of albuminuria [47].

The presence of the Wilms’ tumor 1 (WT1) protein in DM patients’ urinary exosomes and an increase in its expression level with a decline in kidney function suggest that it could be useful as an early noninvasive marker for DN. Patients with proteinuria had significantly higher levels of the urinary exosomal WT1 protein (*p* = 0.001) than those without. Exosomal WT1 levels were linked to an increase in urine protein-to-creatinine ratio (uPCR), albumin-to-creatinine ratio (uACR), and serum creatinine, as well as a decrease in eGFR. Furthermore, patients with WT1-positive urinary exosomes had a lower kidney function than WT1-negative patients. ROC analysis showed that WT-1 effectively predicted GFR < 60 mL/min/1.73 m^2^ (WT-1 protein levels with a cut-off value of 1.9, area under the curve (AUC): 0.92, sensitivity: 88.6%, and specificity: 100%) [48]. However, another group of proteins in urinary exosomes has shown an association with DN: for example, C-megalin (type 2 DN) [49], epithelium-specific transcription factor-3 (type 2 DN) [50], regucalcin [33], osteoprotegerin [51], and CD63 [52] (Figure 1B). As an example, the presence of the urinary exosomal Elf3 protein in DN patients suggested the presence of irreversible podocyte injuries. The rate of decline in eGFR following the measurement of urinary exosomal Elf3 protein concentrations in DN patients (R^2^ = 0.7259) might be useful as an early noninvasive marker for podocyte injuries in DN [50].

In a proteomic quantitative analysis on urine samples from DN patients in advanced disease stages (CKD stages III–V), a panel of three potential proteins was included (protein fragment of alpha-1-microglobulin/bikunin precursor (AMBP), isoform 1 of histone-lysine N-methyltransferase (MLL3), and voltage-dependent anion-selective channel protein 1 (VDAC1)] [53]. Urinary AMBP levels were found to be elevated in DN exosomes in particular. This secreted protein is a membrane glycoprotein with serine protease inhibitor activity that is expressed by the liver and kidney [54]. Increased urine exosomal AMBP could represent increased liver production of ABPM that is filtered from the blood and attached to exosomes in the urinary space, local AMBP synthesis triggered by the disease milieu, or selective incorporation of AMBP into exosomes as a pathologic response mechanism. Besides, MLL3 was only detected in DN urinary exosomes [53]. MLL3 is a critical H3 lysine 4 (H3K4) methyltransferase (H3K4MT) for peroxisome proliferator-activated receptor (PPAR)-dependent adipogenesis, raising the possibility that specific agonists/antagonists of MLL3/4 H3K4MT activity could be useful for treating a variety of metabolic disorders [55]. Finally, VDAC1 levels in urinary exosomes isolated from DN samples were reduced. The reduced exosomal presence of VDAC1 in DN urine may be related to the regulation of apoptosis via exosomal secretion of this protein. VDAC1′s presence in most cell types may limit its utility as a selective marker of kidney cell injury in DN [53].

In a small Chinese study, podocalyxin concentrations in urinary extracellular vesicles were significantly higher in DN patients, indicating the utility of podocalyxin as a potential marker for clinical diagnosis of diabetic nephropathy [56]. Besides, the association between microRNAs (miRNAs) in urinary exosomes and DN was reviewed by Sinha et al. [57]. The following distinct exosomal miRNA expression might provide a prognostic tool for DN: miR-877-3p, miR-362-3p, miR-150-5p, miR-15a-5p (type 2 DN) [58], miR-320c, miR-6068, miR-1234-5p, miR-6133, miR-4270, miR-4739, miR-371b-5p, miR-638, miR-572, miR-1227-5p, miR-6126, miR-1915-5p, miR-4778-5p, miR-2861, miR-30d-5p, miR-30-e-5p (type 2 DN) [59], miR-155, miR-424, miR-130a, miR-145 (type 2 DN) [41], let-7c-5p, miR-29c-5p, miR-15b-5p (type 2 DN) [60], miR-19b-3p (type 2 DN) [61], miR-133b, miR-342, miR-30a (type 2 DN) [62], miR-15b, miR-34a, miR-636, miR-133, miR-342, and miR-30a (type 2 DN) (Table 1) [63].

**Table 1:** Overview of studies of urinary extracellular vesicles in diabetic nephropathy. ZBF: Zucker diabetic fatty rats; HCs: healthy controls; LC–MS/MS: liquid chromatography–tandem mass spectrometry; DM: diabetes mellitus; DN: diabetic nephropathy; AQP1: aquaporin 1; AQP2: aquaporin 2; CCL21: chemokine ligand 21; eGFR: estimated glomerular filtration rate; CI: confidence interval; ROC: receiver operating characteristic curve; AUC: area under the curve; uACR: urine albumin-to-creatinine ratio; WT1: Wilms’ tumor 1; sCr: serum creatinine; NA: normoalbuminuria (<30 mg/g creatinine); MIA: microalbuminuria (30–300 mg/g creatinine): MAA: macroalbuminuria (>300 mg/g creatinine); MCN: minimal change nephropathy; BMP4: bone morphogenetic protein 4; Elf3: epithelium-specific transcription factor-3; STZ: streptozotocin; CKD: chronic kidney disease; OPG: osteoprotegerin; EM: electron microscopy; AMBP: alpha-1-microglobulin/bikunin precursor; VDAC1: voltage-dependent anion-selective channel 1; MLL3: mixed-lineage leukemia protein 3; OPG: osteoprotegerin; SRM: selected reaction monitoring; ue-DPPIV: urinary excretion of microvesicle-dipeptidyl peptidase-IV; MN: membranous nephropathy; FSGS: focal segmental glomerulosclerosis; TSG 101: tumor susceptibility gene 101; PCX: pyruvate carboxylase.

Study Design	Study Population	Technique	Major Findings	Reference
Cross-sectional study Cross-sectional study Cross-sectional study	7 ZBF rats and 7 HC rats STZ-induced diabetic rats 4DM patients with CKD vs. 3 HCs Humans: 12 NA DN patients vs. 12 MIA DN patients vs. 10 HCs Animals: 5 male C57BL6/J STZ-induced diabetic mice vs. 5 male C57BL6/J mice	LC-MS/MS Western blot Western blot qRT-PCR qRT-PCR	286 proteins with functions involving transport, signaling and cellular adhesions were identified and quantified. STZ- induced rats: calnexin and regucalcin: not detected Humans: calnexin and regucalcin not detected Humans: miR-155 and miR-424 were significantly lower in MIA compared to NA DN patients, whereas miR-130a, miR-145 and miR-145 MIA were significantly higher (*p* < 0.05) Animals: urinary exosomes were significantly more present in DM patients compared to healthy control rats (*p* < 0.05).	[32] [33] [41]
Cross-sectional study	4 DM patients vs. 4 biopsy-proven DN patients vs. 4 HCs	Western blot	Significant different levels of AQP1, AQP2 and CCL21 mRNA were found in DN patients compared to HCs (*p* < 0.05). Significant correlations were discovered between CCL21 mRNA and 24h proteinuria and eGFR (r = 0.8009, *p* < 0.0001; r = −0.5186, *p* = 0.0160, respectively). ROC-AUC analysis revealed an excellent diagnostic accuracy of CCL21 mRNA in distinction of both DN from DM patients and incipient DN patients from overt DN patients (AUC = 0.888, 95% CI 0.752–1; AUC: 1.0, 95% CI 1.0–1.0, both *p* < 0.0001).	[46]
Cross-sectional study Cross-sectional study	127 DM2 patients (MIA: *n* = 50, MAA: *n* = 34, NA: *n* = 43) and 34 age- and sex-matched HCs 48 DM1 patients (with uACR > 30 mg/g creatinine: *n* = 18; with uACR < 30 mg/g creatinine: *n* = 30) vs. 25 HCs	ELISA and Western blot Western blot	Ue-DPPIV were significantly different in NA and MIA patients compared to HC and MAA patients (*p* < 0.01). Significant correlations were discovered between WT1 and uACR, sCR and eGFR (r = 0.89, *p* < 0.001; r = 0.71, *p* < 0.001 and r = −0.62, *p* < 0.001, respectively). The best diagnostic accuracy was found for cut-off values of 1.9 for WT1 for distinction between patients with eGFR < 60 and ≥60 mL/min^−1^/1.73 m^2^ (AUC = 0.92, 95% CI: 0.89–1.01, *p* < 0.0001, sensitivity = 88.6%, specificity = 100%).	[47] [48]
Cross-sectional study	56 DM2 patients and 19 HCs	Western blot	C-megalin levels were significantly different in MIA patients compared to NA and MAA diabetics (*p* < 0.05). Significant correlations were discovered between c-megalin and uACR and eGFR (r = 0.805, *p* < 0.001; r = 0.731, *p* < 0.001, respectively).	[49]
Cross-sectional study	25 DN patients and 25 MCN vs. 5 HCs	Western blot	Cultured podocytes: WT1, glomerular RII expression and Smad3 levels differed significantly in DN patients compared to MCN patients (*p* < 0.05). Elf induction caused a significant alteration in WT1, glomerular RII expression and Smad3 levels (*p* < 0.05).	[50]
Cross-sectional study	10 stable CKD patients vs. 4 HCs	Western blot	OPG in urinary exosomes differed significantly in CKD patients compared to HCs (*p* < 0.05)	[51]
Interventional follow-up study	62 MIA DN patients: 2 groups: group 1: routine treatment (*n* = 29) and group 2: treatment with 600 mg/d α-lipoic acid IV (*n* = 33)	Electron microscopy and flow cytometry	CD63 levels in urinary exosomes differed significantly in NA patients compared to MIA patients (*p* < 0.05).	[52]
Cross- sectional study	Discovery phase: 5 DN patients CKD III-V and 5 HCs Confirmation phase: 3 DN patients CKD III-V and 3 HCs	nLC–MS/MS SRM	Discovery phase: AMBP, VDAC1 and MLL3 differed significantly in DN compared to HCs (*p* < 0.05). Confirmation phase: results of the discovery phase were confirmed by SRM.	[53]
Cross-sectional study	57 DM (uncomplicated DM: *n* =34, DN: *n* = 23) vs. other types of nephropathy (MN, IgA nephropathy and FSGS): *n* = 21) and 11 HCs	Western blot	TSG 101 was present in DN, MN, IgA nephropathy and FSGS patients, whereas PCX was only present in DN patients. PCX levels were significantly different in DN patients compared to HCs, DM, other nephropathy groups (*p* < 0.05).	[56]
Cross-sectional study	10 DM2 patients (NA: *n* = 5; MAA: *n* = 5)	NanoDrop ND-1000 Spectrophotometer qRT-PCR	miR-877-3p, miR-362-3p, miR-150-5p and miR-15a-5p were significantly different in NA patients compared to MAA patients (*p* < 0.001).	[58]
Cross-sectional study	Validation cohort: 8 DN patients vs. 8 DM2 patients vs. 8 HCs Confirmation cohort: 5 DN patients, 6 DM2 patients and 6 HCs	Microarray analysis qRT-PCR	Validation cohort: miR-320c, miR-6068, miR-6133, miR-638, and miR-572 levels were strongly significantly different in DN patients compared with HCs and DM2 patients (*p* < 0.01). For miR-320c, significant correlations were found with eGFR and uACR in DN patients (r = 0.08, *p* = 0.55, r = 0.69, *p* = 0.02). Confirmation cohort: findings for miR-320c were verified.	[59]
Cross-sectional study	28 DN DM2 patients vs. 20 DM2 patients vs. 15 HCs	Western blot	Let-7c-5p levels were significantly higher in DN patients compared to HCs, whereas miR-29c-5p and miR-15b-5p were significantly lower (*p* < 0.05). ROC-AUC analysis revealed good diagnostic accuracies of Let-7c, miR-29c-5p and miR-15b-5p for diagnosing DN (AUC = 0.818, AUC = 0.774 and AUC = 0.818, respectively).	[60]
Cross-sectional study	15 DM2 patients vs. 28 DN DM2 patients	qRT-PCR	MiR-19b-3p levels were significantly different in DN patients compared to type 2 diabetics as in patients with and without tubulointerstitial inflammation (*p* < 0.001).	[61]
Cross-sectional study	166 DM2 patients (NA: *n* = 56, MIA: *n* = 66; MAA: *n* = 44) and 54 HCs	RT-PCR	miR-133b, miR-342 and miR-30a HCs vs. MIA, NA vs. MIA, MIA vs. MAA: *p* < 0.01. The best diagnostic accuracy was found for miR-342 with a cut-off value of 0.879 (sensitivity = 81.8%, specificity = 80.9%, PPV = 81.1%, NPV = 81.7% and accuracy = 85.2%)	[62]
Cross-sectional study	Screening group: 40 DM2 patients (MIA: *n* = 17, MAA: *n* = 9, NA: *n* = 14) vs. 12 HCs Confirmation group: 136 DM2 patients (MIA: *n* = 56, MAA: *n* = 34, NA: *n* = 46) and 44 HCs	PCR array RT-PCR	Screening group: miR-15, miR-34a, miR-636, miR-133, miR-342 and miR-30a MIA and MAA vs. NA and HCs: *p* < 0.05 Confirmation group: confirmation of results in screening group and investigation of diagnostic accuracy of miR-15b, miR-34a and miR-636 (AUC = 0.883-0.984)	[63]

### 2.2. IgA Nephropathy

IgA nephropathy (IgAN) is one of the most common primary kidney diseases in the world, and it is a major cause of ESKD [64]. It is distinguished by the presence of IgA1 deposits in the glomerular mesangium. The clinical manifestations of IgAN patients range from asymptomatic microscopic hematuria to rapidly progressive glomerulonephritis. Although persistent proteinuria can indicate active capillary and podocyte lesions, it cannot distinguish between active inflammation and chronic damage [65,66].

At different stages and in different lesions, inflammatory responses are involved in the pathogenesis of IgAN, possibly through the recruitment and activation of different types of leukocyte populations [67]. Exosome excretion was significantly higher in IgAN patients compared with controls, and it was linked to proteinuria and tubular injury. More importantly, urinary exosome excretion was associated with increased histologic activity (mesangial hypercellularity, crescents, and endocapillary hypercellularity). The increased exosome production could be attributed to the proliferation of renal epithelial cells. The release of exosomes appears to decrease as the injury progresses toward the end stage with kidney fibrosis. Proteinuria may increase exosome release due to lysosome dysfunction in tubular epithelial cells. However, the mechanisms by which proteinuria increases exosome excretion require further investigation [68].

Exosomal chemokine (C-C motif) ligand 2 (CCL2) was found to be upregulated in IgAN patients through mRNA profiling. CCL2 was found to be exclusively highly expressed in IgAN patients compared with healthy controls, minimal change disease, and membrane nephropathy patients in a validation study. In IgAN, there was also a correlation between exosomal CCL2 and eGFR. CCL2 exosomes were linked to tubulointerstitial inflammation and C3 deposition. High CCL2 levels at the time of kidney biopsy were linked to worsening kidney function later on. Urinary exosomes and exosomal CCL2 mRNA are promising biomarkers in IgAN that reflect active kidney histologic injury and kidney function deterioration. The underlying mechanism by which exosomal CCL2 participates in disease pathogenesis will need to be investigated further (Table 2) [68].

Besides, 158 miRNAs in urinary exosomes were differentially expressed between IgAN patients and healthy controls, with 41 miRNAs being significantly and differentially expressed [69]. Among these 41 miRNAs, miR29c, miR146a, and miR205 have been linked to IgAN pathogenesis and progression [70], and may serve as IgAN biomarkers. MiR-215-5p and miR-378i were significantly upregulated (*p* < 0.01) in urinary exosomes of IgAN patients compared with healthy controls, while miR-29c and miR-205-5p were significantly downregulated (*p* < 0.05). MiR-215-5p, miR-378i, miR-365b-3p, and miR-135b-5p were found to have altered expression in validation cohort patients with IgAN [69]. Another study showed that the presence of urinary exosomal miR-4639 was linked to more serious and active histological activity (mesangial hypercellularity, crescent, and C3 complement deposition). MiR-4639 and miR-210 expressions in plasma and urinary exosomes were higher in patients with progressive IgAN after an average of 8 months of follow-up [71]. Additionally, hsa-miR-451a and hsa-let-7d-3p might be used as noninvasive biomarkers to assess IgAN: hsa-miR-451a (AUC = 0.805, *p* = 0.001), hsa-mir-7d-3p (AUC = 0.76, *p* = 0.0049), and the combination of hsa-miR-451a and hsa-let-7d-3p (AUC = 0.8125, *p* = 0.0007). According to the Oxford classification, S0 had lower levels than S1 for hsa-miR-451a (*p* = 0.016), and M0 had lower levels than M1 for hsa-mir-7d-3p (*p* = 0.05) [72]. When IgAN patients were compared with healthy controls, urinary exosomal miR-204 expression was significantly lower. MiR-204 expression did not differ between IgAN and non-IgAN CKD controls. Patients with IgAN who were at high risk of future progression had significantly lower miR-204 expression than those who were at low risk of progression. The AUC for the two IgAN cohorts was 0.82 based on a ROC analysis. Furthermore, miR-204 expression was associated with known clinicopathological prognostic risk factors. Importantly, incorporating miR-204 into the International IgAN Risk Prediction Tool improved the algorithm’s diagnostic power in predicting the risk of progression [73].

**Table 2:** Overview of studies of urinary extracellular vesicles in IgA nephropathy. HCs: healthy controls; MN: membranous nephropathy; MCNS: minimal change nephrotic syndrome; CCL2; chemokine (C-C motif) ligand 2; eGFR: estimated glomerular filtration rate; RT-qPCR: real-time quantitative polymerase chain reaction; MCN: minimal change nephropathy; AUC: area under the curve; IgANnp: IgA nephropathy nonprogressors (∆sCr < 10% over 10 years since diagnosis); IgANp: IgA nephropathy progressors (sCr doubled or developed ESKD over 10 years since diagnosis); TMN: thin membrane nephropathy; NGS: next-generation sequencing.

Study Design	Study Population	Technique	Major Findings	Reference
Cross-sectional study	Screening cohort: 6 IgA patients and 6 HCs Validation cohort: 55 IgA patients vs. 4 HCs vs. 16 MN patients vs. 9 MCNS	Western blot Western blot	Screening cohort: *CCL2* gene: 10-fold induction in IgA patients compared to controls Validation cohort: significant correlations were discovered between level of urinary CCL2 mRNA and eGFRs (r = −0.624, *p* < 0.05)	[68]
Cross-sectional study	Screening cohort: 12 IgA patients vs. 12 HCs Validation cohort: 6 IgA patients vs. 6 HCs	Western Blot RT-qPCR	Screening cohort: miR-215-5p, miR-378i, miR-29c and miR205-5p levels were significantly higher in IgA patients compared to HCs (*p* < 0.01). Validation cohort verified the results of the screening cohort.	[69]
Follow-up study	30 IgA patients vs. 10 MN patients vs. 7 MCN vs 7 DN patients vs. 30 HCs	Western Blot qRT-PCR	Urinary miR-4639 and miR-210 differed significantly in IgA patients compared to MN, DN, MCN and HCs, and miR-4639 and miR-210 were even significantly different between patients with progressive IgAN vs. non-progressive IgAN (*p* < 0.05). Exosomal miR-4639 and miR-210 were linked to eGFR and proteinuria, respectively, in plasma (r = −0.5424, *p* < 0.0001 and r = −0.4801, *p* = 0.0001) and urine (r = 0.7725 and r = 0.6010, both *p* < 0.0001) Plasma exosomal miR-4639 and miR-210 performed better than proteinuria (g/24 h) to predict kidney outcomes (AUC = 0.77 and AUC = 0.79–0.83, respectively).	[71]
Cross-sectional study	20 IgA patients vs. 20 HCs	RT-qPCR	The IgAN group had considerably higher levels of hsa-miR-451a and hsa-let-7d-3p than the HC group (*p* < 0.05). Both the levels of hsa-miR-451a and hsa-let-7d-3p were associated with the severity of the disease. High AUCs for an IgAN diagnosis were present in the exosomes hsamiR-451a and hsa-let-7d-3p (0.805 and 0.76, respectively).	[72]
Cross-sectional study	Screening cohort: 15 IgA patients vs. 8 CKD patients vs. 6 HCs Confirmation cohort: 6 IgANnp vs. 6 IgANp vs. 6 TMN vs. 6 MN	RT-qPCR NGS and RT-qPCR	Screening cohort: comparing IgAN to healthy participants, urinary exosomal miR-204 expression was considerably lower in IgAN (*p* < 0.05). Confirmation cohort: an AUC of 0.82 was found by ROC-analysis comparing the two IgAN cohorts. Moreover, miR-204 expression was significantly correlated with eGFR and proteinuria.	[73]

### 2.3. Lupus Nephritis

Lupus nephritis (LN), which affects 40–75% of systemic lupus erythematosus patients, is one of the most severe forms of the disease and has an unpredictable course. Despite modern therapeutic approaches, LN remains a significant cause of short- and long-term morbidity [74], with up to 20% of patients progressing to ESKD [75]. Routine clinical parameters are currently insufficiently sensitive or specific for detecting ongoing disease activity and progression, early relapse, or response to therapy [76].

Several exosomal-derived miRNAs have been identified in LN as markers of early fibrosis [77,78], podocyte injury [79], type IV nephritis [80], and the presence of cellular crescents [81], as well as being able to distinguish active LN [82]. Urinary miR-31-5p, miR-107, and miR-135b-5p levels were higher in responder patients during flare than in nonresponders. The majority of miRNA expression was found in epithelial tubular cells, but miR-135b-5p was also found in the glomeruli. The study of potential recipient cells for these miRNAs revealed that responder urinary-labeled exosomes were taken up significantly more by endothelial cells in the early stages, and mesangial cells over time. This research suggests that these two cells could be miRNA targets and play a role in kidney repair. Levels remained elevated for at least a year after treatment, implying that these miRNAs may play a role in kidney recovery [83]. Hypoxia-inducible factor-1 alpha (HIF-1α) has been identified as a common target of these miRNAs [84,85,86]. HIF-1α downregulation was enhanced by the simultaneous overexpression of miR-31-5p, miR-107, and miR-135b-5p. HIF-1α downregulation may aid kidney recovery by inhibiting mesangial cell proliferation and decreasing the expression of mesangial inflammatory chemokines (CXCL1, CCL3, and CCL2) and interleukin-6 (IL-6). Furthermore, by reducing the expression of IL-6 and vascular cell adhesion molecule 1 (VCAM-1) in endothelial kidney cells, it may contribute to kidney recovery. The study of miRNAs had the ability to discriminate between responders and nonresponders, according to ROC curve analysis, with miR-135b-5p having the best predictive profile, with a sensitivity of 77.8% and a specificity of 71.4% to discriminate clinical response. Correlation analysis with clinical parameters revealed a link between urinary exosome miR-135b-5p expression levels and proteinuria, and miR-31 levels were found to be inversely related to kidney activity score in the responder group. The identification of relevant miRNAs involved in LN kidney recovery may aid in the development of new therapeutic approaches [83]. Urinary exosomal miR-195-5p, miR-25-3p, and miR-429 were all downregulated in patients, and miR-195-5p was able to distinguish LN patients from SLE with high sensitivity and specificity, indicating a promising future for LN disease monitoring and diagnosis [87]. Let-7a and miR-21, two miRs known to be differentially expressed in LN tissues and/or serum, were downregulated in urinary exosomes during an active flare of LN [88]. Urinary exosomal miR-146a levels were inversely related to circulating complement C3 and C4 levels, proteinuria, and the chronicity index in a kidney biopsy. Higher basal miR-146a levels in urinary exosomes were linked to disease activity and could predict flares up to 36 months later. MiR-146a in urinary exosomes may represent a protective mechanism against nuclear factor-B (NF-B) and interferon (IFN)-mediated podocyte inflammation and injury, according to in vitro studies [89]. In another study, elevated miR-654-5p and miR-3135b levels in urinary exosomes distinguished class IV LN patients with cellular crescents from those without [81]. The precise roles of these miRs in glomerular inflammation are unknown, as their role in LN pathogenesis has not previously been described [90]. Urinary exosomal miR-29c was found to be negatively correlated with the histological chronicity index and glomerular sclerosis in the context of LN [77]. Exosomal miR-150 was the most sensitive and specific in distinguishing between groups with high and low chronicity index. This supports the previously described role of miR-150 in mediating kidney fibrosis in LN kidney tissue. When urinary exosomal miR-29c and miR-21, two other miRs involved in the regulation of the TGF-β1/Smad3 pathway and implicated in other kidney diseases, were added to miR-150, the specificity for diagnosing early kidney fibrosis increased even more [78]. A panel of miRs derived from urinary exosomes was proposed to diagnose early kidney fibrosis and predict progression to ESKD (Table 3) [90].

**Table 3:** Overview of studies of urinary extracellular vesicles in lupus nephritis and ANCA-associated vasculitis. LN: lupus nephritis; HCs: healthy controls; RT-qPCR: real-time quantitative polymerase chain reaction; CIn: chronicity index; GN: glomerulonephritis; LN-IV: class IV lupus nephritis; CC: cellular crescent; SLE: systemic lupus erythematosus; AUC: area under the curve; ANCA: antineutrophil cytoplasmic antibodies; IL: interleukin; TNF-α: tumor necrosis factor-alpha; IFN: interferon; HIF-1α: hypoxia-inducible factor-1 alpha; VCAM-1: vascular cell adhesion molecule 1; CCL2: **c**hemokine (C-C motif) ligand 2; CCL3: **c**hemokine (C-C motif) ligand 3; LPS: lipopolysaccharide; TRAF6; TNF receptor-associated factor 6; IRAK1: interleukin receptor-associated kinase 1; AAV: ANCA-associated vasculitis; uEV: urinary extracellular vesicles; MAN1A1: mannosidase 1A1; MCP-1: monocyte chemoattractant protein-1.

Pathology	Study Design	Study Population	Technique	Major Findings	Reference
LN	Cross-sectional study	45 LN patients and 20 HCs	RT-qPCR	Urinary exosomal miR-21, miR-150, and miR-29c were linked with LN CIn (r = 0.565, 0.840, and 0.559, respectively). In LN patients, this miRNA profile discriminated low CIn from moderate-high CIn with a high degree of specificity (94.4%) and sensitivity (99.8%).	[78]
	Cross-sectional study	Animals: female B6.MRLc1 (GN) and C57BL/6 mice (HCs) Humans: 13 LN vs. 8 HCs	RT-qPCR RT-qPCR	Mice: in mice glomeruli and podocyte cell lines, miR-26a is selectively expressed at high levels. Humans: MiR-26a levels in urine exosomes were significantly higher in LN patients than in healthy controls (*p* < 0.05), and were positively linked with proteinuria (r = 0.696, *p* < 0.01).	[79]
	Cross-sectional study	4 LN-IV vs. 10 SLE without LN (LNN group) and 7 patients without AI disease (control)	Illumina sequencing	First-time associations between 14 novel microRNAs and LN have been made (hsa-miR-589-3p, hsa-miR-1260b, hsa-miR-4511, hsa-miR-485-5p, hsa-miR-584-5p, hsa-miR-543, hsa-miR-153-3p, hsa-miR-6087, hsa-miR-3942-5p, hsa-miR-7977, hsa-miR-323b-3p, hsa-miR-4732-3p and hsa-miR-6741-3p, all *p* < 0.05). MiR-107-3p was also discovered to be 5.9 more numerous in LN-IV patients than in healthy individuals.	[80]
	Cross-sectional study	44 LN patients (active LN-IV: *n* = 15; LV-IV CC: *n* =14 and inactive LN-IV: *n* = 14)	Identification technique: Western blot Validation technique: RT-qPCR	Identification phase: between the LN-IV CC and LN-IV (active and inactive) groups, 66 changed exosomal miRNAs with statistical significance were found (*p* < 0.05). Validation phase: LN-IV-CC differed from other groups in the urine exosome miRNA expression pattern, and miR-3135b and miR-654-5p were validated as possible LN-IV-CC biomarkers. The specificity of their forecast ranged from 83.33 to 96.67%.	[81]
	Cross-sectional study	38 SLE patients (6 active LN, 10 inactive LN and 12 SLE without L) vs. 12 HCs	RT-qPCR	Active and inactive LN might be distinguishable by miR-146a with an ideal threshold value of 47.2-fold change resulting in an AUC of 0.867 (*p* < 0.05, sensitivity = 80% and specificity = 89%).	[82]
	Cross-sectional study	43 proliferative LN patients (clinical response: *n* = 22; no clinical response: *n* = 21)	qRT-PCR	In urine and kidney tissue, responders had significantly higher levels of miR-31, miR-107, and miR-135b-5p than non-responders (*p* < 0.05), with miR-135b having the strongest predictive value for differentiating responders (AUC = 0.783, sensitivity = 77.8% and specificity = 71.4%). In vitro analysis revealed that tubular cells treated with inflammatory cytokines (such as IL-1, TNF-α, IFN, and IL-6) are the main source of exosome-derived miR-31, miR-107, and miR-135b-5p production. Mesangial cells from responders were better at absorbing urinary exosomes (90% vs. 50%, *p* < 0.0001) than mesangial cells from non-responders. Finally, HIF-1α suppression decreased endothelial cell production of IL-6/VCAM-1, mesangial cell production of IL-8, CCL2, CCL3, as well as mesangial cell proliferation.	[83]
	Cross-sectional study	47 SLE patients (SLE with LN: *n* = 26, SLE without LN: *n* = 21) vs. 20 HCs	RT-qPCR	LN patients’ urine exosomal miR-195-5p, miR-25-3p, and miR-429 levels were significantly decreased (*p* < 0.05), and miR-195-5p had a good discriminatory power in differentiating LN from SLE patients with an AUC of 0.89.	[87]
	Cross-sectional study	13 active LN patients vs. 18 inactive LN patients	qPCR	Let-7a and miR-21 levels were considerably lower in patients with active disease (*p* < 0.05).	[88]
	Cross-sectional study	41 SLE patients (with LN: *n* = 27, without LN: *n* = 14) vs. 20 HCs	RT-qPCR	Exosomal miR-146a was found to be strongly associated with changes in proteinuria and lupus activity. Exosomal miR-146a was able to identify LN patients with an AUC of 0.81 (sensitivity = 67%, specificity = 88%, *p* < 0.001) and detected flares in LN patients with an AUC of 0.88 ± 0.055 (*p* < 0.0001). An in vitro examination revealed that LPS stimulation caused a significant increase of miR-146a levels, TRAF6 and IRAK1 mRNA expression (*p* < 0.05).	[89]
ANCA-associated vasculitis	Cross-sectional study	24 AAV patients vs. 16 HCs	qRT-PCR	In the uEVs of AAV patients, 5 miRNAs (miR-30a-5p, miR-31-3p, miR-99a-5p, miR-106b-5p, and miR-182-5p) were significantly increased (*p* < 0.05).	[91]
	Cross-sectional study	Test cohort: 10 AAV patients vs. 10 HCs Validation cohort: 10 AAV patients vs. 10 IgA patients vs. 10 HCs	LC-MS/MS Microarray analysis	Test cohort: 475 statistically significant differentially altered proteins between healthy donors and AAV patients in uEV samples were identified (*p* = 0.05). Validation cohort: the antibody microarray assay confirmed significant changes in protein levels of MAN1A1, haptoglobin, nidogen-1 and MCP-1.	[92]

### 2.4. ANCA-Associated Vasculitis

Antineutrophil cytoplasmic antibody (ANCA)–associated vasculitis (AAV) is a high-mortality autoimmunity. The pathophysiological mechanisms underlying complex AAV pathogenesis are largely unknown. The content of EVs found in urine can be analyzed molecularly to gain new insights into this extremely complex issue [91,92].

Five miRNAs (miR-30a-5p, miR-31-3p, miR-99a-5p, miR-106b-5p, and miR-182-5p) found in high concentrations in uEVs have the potential to regulate cell surface processes and signaling events, as well as act in concert with some differentially expressed proteins in uEVs [91]. The miRNAs should be investigated further to determine their roles in AAV pathogenesis and their association with disease activity and progression (Table 3).

### 2.5. Idiopathic Membranous Nephropathy

Membranous nephropathy is a group of diseases characterized by immune complex deposition under glomerular basement membrane epithelial cells and basement membrane thickening. In terms of etiology, it is divided into idiopathic membranous nephropathy (unknown etiology) and secondary membranous nephropathy [13]. Idiopathic membranous nephropathy is an antibody-mediated kidney disease in which IgG autoantibodies from subepithelial immune complexes with autoantigens are expressed on the cell surface of podocytes [93].

There are significant differences in 25 urinary exosome miRNA profiles between idiopathic membranous nephropathy (IMN) patients and healthy people. MiR-9-5p and miR-30b-5p were chosen for verification, and the results matched those obtained through high-throughput sequencing. MiR-9-5p was found to be related to triglyceride levels and eGFR. MiR-30b-5p levels were linked to anti-phospholipase A2 receptor antibody, serum albumin, α2-microglobulin, and the global sclerosis/observed glomeruli number ratio. The analysis of ROC curves revealed that miR-30b-5p and miR-9-5p could be used to detect IMN [13]. MiR9-5p, miR-30b-5p, miR-532-3p, miR-429, miR-129-5p, miR-107, miR-25-3p, and miR-206 were found to be involved in podocyte injury in the differential expression profile. MiR-532-3p, miR-9-5p, miR-30b-5p, miR129-5p, miR-125b, and miR-338-5p have all been linked to regulatory T cells (Tregs) regulation. MiR-30b-5p and miR-9-5p may play a role in the pathogenesis of IMN, more specifically to kidney fibrosis by preventing the downregulation of genes related to key metabolic pathways, including mitochondrial function, oxidative phosphorylation, fatty acid oxidation, and glycolysis in unilateral ureteral obstruction mice [94]. MiR-9-5p is downregulated in IMN patients’ urinary exosome miRNAs, which may reflect active metabolism of kidney fibrosis-related pathways. miR-9-5p and miR-30s may also be involved in podocyte homeostasis maintenance [13].

Another small Chinese study with six patients with an IMN and five control subjects showed that 108 miRNAs were differentially coexpressed between the two groups [95], of which several are associated with kidney diseases. MiR-9-5p confers protection against CKD and kidney fibrosis [94]. MiR-92b-3p was involved in the development of kidney abnormalities caused by advanced glycation end products (AGEs) [96]. MiR-145-5p inhibited high glucose-induced apoptosis by targeting Notch1 and then dysregulating apoptotic factors [97]. The phosphatidylinositol 3-kinases/protein kinase B (PI3K/AKT) signaling pathway may be used by miR-27b to inhibit angiogenesis and fibroblast activation [98]. MiR-615-3p improved splenic macrophage phagocytic potential by targeting the ligand-dependent nuclear receptor corepressor [99]. MiR-197-3p was primarily involved in signaling cascades that result in cytokine production [100,101].

Besides, lysosome membrane protein-2 (LIMP-2), also known as scavenger receptor class B, member 2 (SCARB2) or LGP85, was found to be more than twice as abundant in urinary microvesicles from IMN patients compared with idiopathic FSGS and normal controls. Along the glomerular basement membrane, LIMP-2 expression was closely associated with IgG. Autoantibodies against LIMP-2 were not found, implying that increased glomerular LIMP-2 expression is caused by other antibodies, such as anti-PLA2R [102]. LIMP-2 may regulate the balance of vesicle invagination and vesicle budding from endosomal compartment limiting membranes, and thus play a role in the biogenesis and maintenance of endosomes and lysosomes [103]. It is possible that the increased expression of LIMP-2 in IMN indicates a general dysfunction in protein transport between endosomes/lysosomes and podocyte plasma membranes (Table 4) [102].

### 2.6. Podocytopathies—Focal Segmental Glomerulosclerosis and Minimal Change Disease

Focal segmental glomerulosclerosis (FSGS) is not a single illness, but rather a collection of distinct clinicopathological conditions that share injury to the podocyte as their fundamental pathophysiological characteristic. The pattern of glomerular sclerosis is segmental, affecting a portion of the glomerular tuft, and localized early in the disease, affecting a small percentage of glomeruli. As the illness worsens, glomerulosclerosis becomes more widespread and diffuse. The typical segmental structure of the sclerosing lesions in the glomerular tuft, until the entire glomerulus is afflicted, may be explained by cell-to-cell propagation of podocyte injury [104,105].

Minimal change disease (MCD) is the most frequent cause of nephrotic syndrome, accounting for 70–90% of patients. However, by adolescence, this proportion drastically reduces as other glomerular diseases, such as MN, become more prevalent [106].

A comparison of FSGS and MCD revealed 155 differentially expressed miRNAs in urine. A comparison analysis showed that a significant number of miRNAs were downregulated in urine samples from FSGS patients compared with MCD patients. Urine mir-1225-5p concentrations were higher in MCD patients compared with FSGS patients and controls (*p* < 0.001). Urinary levels of mir-1915 and miR-663 were lower in FSGS patients compared with MCD and controls (*p* < 0.001), whereas urinary levels of miR-155, an inflammation-related miRNA, were higher in FSGS patients compared with MCD and controls (*p* < 0.005) (Table 4). Further research is needed to determine the cause and effect of miRNA expression in FSGS [107].

### 2.7. Autosomal Dominant Polycystic Kidney Disease

Autosomal dominant polycystic kidney disease (ADPKD) is the most common monogenic-inherited kidney disease, caused primarily by mutations in either *PKD1* (85%) or *PKD2* (15%), with an estimated incidence of ~1 in 1000. ADPKD is distinguished clinically by progressive cyst formation and marked kidney enlargement caused by the sustained expansion of multiple fluid-filled cysts. Cystic progression causes crowding of neighboring nephrons, resulting in injury to normal-functioning parenchyma and loss of kidney function [108].

Secreted EVs/exosomes regulate the biology and function of neighboring cells, such as renal epithelial cells, fibroblasts, and macrophages, and contribute to the formation of kidney cysts. The roles of EVs/exosomes in ADPKD are not yet completely understood, as are the in vivo mechanisms underlying the EVs/exosome-elicited action. EVs/exosomes may be secreted not only from the apical sides of cystic renal epithelial cells to cyst fluid, but also from the apical and basolateral sites of cystic renal epithelial cells. According to a “cystic EVs/exosomes theory”, cystic cell-derived EVs/exosomes are one of the key players in the pathogenesis of ADPKD. EVs/exosomes derived from cystic cells and urinary exosomes derived from ADPKD patients not only affected recipient cell function but also promoted cyst growth in PKD1 mutant mice and 3D cultures by downregulating *PKD1* gene expression and upregulating miRNAs, including miR-200s and miR-21, leading to the activation of PKD-associated signaling pathways. Treatment with cystic cell-derived EVs/exosomes induced: (1) fibroblast activation and the expression of fibrotic markers, which increased kidney fibrosis, and (2) cytokine expression and macrophage recruitment, which increased kidney inflammation in cystic kidneys. Cystic cell-derived EVs/exosomes may influence the biology and function of recipient cells, thereby promoting cyst progression. A positive feedback loop between miRNAs in cystic cells and miRNAs in EVs/exosomes and recipient cells could be one of the mechanisms by which cystic cell-derived EVs/exosomes promote cyst growth, kidney inflammation, and kidney fibrosis. The expression of genes associated with EVs/exosome biogenesis was upregulated in PKD1 mutant renal epithelial cells and tissues, implying that cystic renal epithelial cell EVs/exosomes may be secreted abnormally. EVs/exosomes may be a nongenetic factor that reduces polycystin-1 levels in cystic kidneys. Exosome biogenesis/release inhibition with GW4869 slowed cyst growth in aggressive and milder ADPKD mouse models. Through secreted EVs/exosomes, PKD mutant renal epithelial cells may affect the cell function of neighboring cells that are not in direct contact with cells, implying a therapeutic potential for ADPKD treatment by targeting abnormal exosome secretion. Future research is needed to identify other mechanisms mediated in recipient cells by cystic cell-derived EVs/exosomes [109].

In comparison with healthy subjects and non-ADPKD CKD patients, cytoskeletal proteins (villin-1 and plakins) and complement-related proteins (C3 and C9) were identified as disease-associated proteins in ADPKD by the proteomic analysis of uEVs. Only uEVs from patients with advanced stages of ADPKD had increased levels of villin-1, periplakin, and envoplakin, whereas uEVs from young patients with ADPKD and preserved kidney function already had higher levels of complement [110]. The discovery of villin-1, plakins, and complement in uEVs from ADPKD patients may be biologically plausible. Villin-1 is an actin-modifying protein that is involved in cell morphology, actin reorganization, and cell motility. It is mostly expressed in the brush border of the proximal tubules in the kidney [111]. Polycystin-1 plays a role in the organization of the actin cytoskeleton, migration, and cell adhesion [112]. Defects in polycystin-1 cause cell polarity defects [113] and abnormal cell growth [114], which could explain the rise in villin-1. The desmosomal plaque is made up of several transmembrane proteins from the cadherin family (also called plakins) [115]. Polycystin-1 no longer colocalizes with desmosomes in ADPKD, resulting in desmosomal protein mispolarization from the basolateral to the apical domain [116]. This could explain why more plakins were detected in the uEVs of ADPKD patients [110]. Additionally, complement activation has previously been linked to ADPKD and autosomal recessive polycystic kidney disease [117,118]. The increased abundance of complement system components in uEVs of ADPKD patients may reflect increased production by kidney cyst epithelial cells [110].

Human urinary exosome miRNA was studied using global small RNA sequencing in a discovery cohort of seven patients with ADPKD with early disease (eGFR > 60 mL/min/1.73 m^2^) and nine with late disease (eGFR < 60 mL/min/1.73 m²), and their differential expression was compared with six age- and sex-matched healthy controls. In patient urine exosomes, murine PKD1 cystic kidneys, and human PKD1 cystic kidney tissue, miR-192-5p, miR-194-5p, miR-30a-5p, miR-30d-5p, and miR-30e-5p were significantly downregulated. All five miRNAs correlated with baseline eGFR and ultrasound-determined mean kidney length, and they improved the AUC of mean kidney length for the rate of disease progression. Finally, inverse correlations between these two miRNA families and increased expression of their predicted target genes in PKD1 cystic tissue revealed dysregulated pathways and transcriptional networks, including novel interactions between miR-194-5p and two potentially relevant candidate genes, *PIK3R1* and *ANO1* (Table 4) [119].

### 2.8. Medullary Sponge Kidney Disease

Medullary sponge kidney disease is a rare congenital malformation characterized by collecting duct dilation in the kidney papillae, urinary acidification and concentration defects, cystic precalyceal duct anomalies associated with a high risk of nephrocalcinosis, and recurrent kidney stones [120].

It has been demonstrated that patients with medullary sponge kidney have a different proteomic profile of urinary microvesicles and exosomes from patients with ADPKD. Proteins involved in cell proliferation and matrix remodeling were detected in the urine proteomic profiles of patients with ADPKD. Proteins found in patients with medullary sponge kidney were linked to parenchymal calcium deposition/nephrolithiasis and systemic metabolic derangements linked to stone formation and bone mineralization defects [121].

Several kinases were able to distinguish medullary sponge kidney disease from idiopathic calcium nephrolithiasis, and three ephrin receptors (EphA1, EphB3, and EphB6) were found to be the most significantly downregulated proteins in medullary sponge kidney disease versus idiopathic calcium nephrolithiasis. Several biological elements, including laminins (primarily laminin 2), ephrins, and other kinases (e.g., MAPK), could orchestrate cell proliferation, cystic dilations of precalyceal ducts, and extracellular matrix remodeling that are typical of this disease (Table 4) [122].

**Table 4:** Overview of studies of urinary vesicles in idiopathic membranous nephropathy, focal segmental glomerulosclerosis, ADPKD, and medullary sponge kidney disease. IMN: idiopathic membranous nephropathy; HCs: healthy controls; RT-qPCR: real-time quantitative polymerase chain reaction; eGFR: estimated glomerular filtration rate; Anti-PLA2R: anti-phospholipase A2 receptor antibody; B2M: β2-microglobulin; GS/GN: global sclerosis/observed glomeruli number; ROC: receiver operating characteristics curve; AUC: area under the curve; FSGS: focal segmental glomerulosclerosis; MCD: minimal change disease; ADPKD: autosomal dominant polycystic kidney disease; CKD: chronic kidney disease; LC–MS/MS: liquid chromatography–tandem mass spectrometry; uEV: urinary extracellular vesicles; MSKD: medullary sponge kidney disease; MS: mass spectrometry.

Pathology	Study Design	Study Population	Technique	Major Findings	Reference
IMN	Cross-sectional study	Test cohort: 6 IMN patients vs. 6 HCs Validation cohort: 30 IMN patients vs. 30 HCs	High-throughput sequencing RT-qPCR	Test cohort: 25 miRNAs were significantly downregulated in IMN patients (*p* < 0.05). Validation cohort: significant correlations between miR-9-5p and triglyceride levels and eGFR, whereas miR-30b-5p revealed a significant relationship with the levels of anti-PLA2R, serum albumin, B2M, and the ratio of GS/GN. According to ROC analysis, exosomal miR-30b-5p and miR-9-5p also seemed to have a good diagnostic value in IMN patients (*p* < 0.05, AUC = 0.867 and 0.724, respectively).	[13]
	Cross-sectional study	6 IMN patients and 5 HCs	Agilent 2200 Bioanalyzer	In the IMN and HC groups, there were 108 differentially co-expressed miRNAs, of which 95 showed an upregulation and 13 showed a downregulation.	[95]
FSGS	Cross-sectional study	16 primary FSGS patients vs. 5 MCD patients vs. 5 HCs	qRT-PCR	FSGS and MCD comparison analyses identified 126 and 155 miRNAs that were differently expressed in plasma and urine. Compared to FSGS patients and healthy individuals, urinary miR-1225-5p levels were considerably higher in MCD patients. While FSGS patients’ urinary levels of miR-1915, miR-663, and miR-155 were significantly different from those of MCD patients and healthy controls (*p* < 0.05).	[107]
ADPKD	Cross-sectional study	Identification cohort: 6 ADPKD (CKD 2-3) patients vs. 6 HCs Confirmation cohort 1: 6 CKD 3 stage patients vs. 6 ADPKD (CKD 2-3) patients vs. 6 HCs Confirmation cohort 2: 4 CKD stage 3 patients vs. 5 ADPKD patients (CKD 2-3) Confirmation cohort 3: 6 ADPKD (CKD 1) patients vs. 11 ADPKD (CKD 2-4) vs. 4 HCs	LC-MS/MS	Identification cohort: several plakins, complement-related proteins, and glycoproteins were more abundant in uEVs of ADPKD patients whereas annexin A2, contactin-1, syndecan-4, and granulins were less abundant in ADPKD patients. Confirmation cohort 1-3: the abundance of villin-1, envoplakin, periplakin, complement 3, and complement 9 was higher in uEVs of ADPKD patients. However, while complement was already increased in uEVs from ADPKD patients with intact kidney function, villin-1, periplakin, and envoplakin were only elevated in progressing CKD.	[110]
	Cross-sectional study	Discovery cohort: 7 ADPKD with early disease (eGFR > 60 mL/ min/1.73 m²) vs. 9 with late disease (eGFR < 60 mL/min/ 1.73 m²) vs. 6 age-and sex-matched HCs Validation cohort: 20 ADPKD patients with early disease vs. 20 ADPKD patients with late disease vs. 20 HCs CKD cohort: 20 CKD patients with DM2 vs. 10 HCs	RNA sequencing qPCR qPCR	Discovery cohort: By comparing the three groups’ differentially expressed miRNAs, a total of 23 miRNAs were found to be significantly different (*p* < 0.05). Validation cohort: when compared to HCs, expression of all 5 miRNAs (miR-192-5p, miR-194-5p, miR-30a-5p, miR-30d-5p and miR-30e-5p) was markedly decreased in ADPKD patients who had advanced illness. Only miR-192- 5p showed a substantial reduction in early disease patients as compared to HCs (fold change 0.54) CKD cohort: although they did not approach statistical significance, alterations for these 5 miRNAs demonstrated a different pattern from ADPKD patients.	[119]
Medullary sponge kidney disease	Cross-sectional study	15 ADPKD patients vs. 15 MSKD patients vs. 17 HCs	MS	2950 proteins in total were isolated from microvesicles and exosomes, of which 1579 (54%) were found to be present in all samples, but only 178 (6%) and 88 (3%) were found to be unique to medullary sponge kidney microvesicles and exosomes, and 183 (6%) and 98 (3%) were found to be unique to ADPKD microvesicles and exosomes, respectively.	[121]

## 3. Kidney Transplantation

With increasing age of living donors, the number of exosomes, juxtaglomerular cells, and podocyte marker-positive EVs decreases [123]. uEV markers after transplantation can be classified into kidney-specific, donor-specific, and immune response-related markers based on their differences. Several studies have demonstrated that kidney-specific markers (podocalyxin-like protein 1 (PODXL), ion cotransporters, SYT17, neutrophil gelatinase-associated lipocalin (NGAL), and CD133) and immune response-related markers (CD3, multi-mRNA signatures, and viral miRNA) can be used to diagnose rejection, BK virus-associated nephropathy, and calcineurin inhibitor nephrotoxicity after kidney transplantation. Furthermore, some indirect evidence for donor-specific markers (donor-derived cell-free DNA) in urine has been demonstrated [124]. We will illustrate the potential value of uEVs in the kidney transplantation setting with some examples below.

A pilot study showed that minor differences in uEVs’ profile could not distinguish between living and deceased donors in normofunctional grafts [125]. A concise atlas reflecting the temporal changes in the proteome of small uEVs during the course of living donor kidney transplantation was assembled and analyzed using differential (ultra) centrifugation. An unbiased proteomic analysis detected over 1700 proteins enriched by the separation protocol. The small uEV proteome was found to form timepoint-dependent subclusters. When the top 20 proteins in terms of abundance were considered, 5 proteins were detectable to a high degree at all timepoints: uromodulin, albumin, complement factor 3, α-1-microglobulin, and cytoplasmic actin. All early timepoints showed an over-representation of proteins expected in serum rather than urine, including fibrinogen chains α and β and apolipoprotein A1 and A4 [126], which may reflect differences in EV packing and a decreased intercellular barrier upon reperfusion, resulting in plasma extracellular vesicles crossing into the urinary space [127,128]. The fact that these proteins can still be detected 4 weeks after transplantation suggests that they are derived from nephron cells. Specific temporal profiles, such as an increase in complement-associated proteins, were observed shortly after transplantation, indicating early immunologic effects. The increase in proteins related to complement activation, regulation, and cytolysis most likely reflects the initial immune response triggered by both ischemia/reperfusion and allograft exposure [126]. This supports the hypothesis that early inhibition of these responses, most likely focusing on the complement system, may be beneficial [129,130]. Furthermore, the abundance of phosphoenolpyruvate carboxykinase (PCK2) in the suEV proteome 1 day after transplantation was found to be predictive of overall kidney function 1 year later. This study demonstrated the utility of analyzing suEVs for monitoring immune response activity and biomarker discovery by employing a quick and simple protocol for their separation [126].

mRNA extracted from urinary exosomes for biomarkers of kidney injury may reflect or predict levels of the corresponding protein after transplantation and clinical outcomes. In a cross-sectional study with kidney transplant patients, healthy subjects, and CKD patients, exosomal mRNA expression for the injury biomarkers NGAL, IL-18, kidney injury molecule-1 (KIM-1), and cystatin C was compared with urinary protein, 18S RNA, and serum creatinine concentrations. Urinary NGAL and IL-18 levels after kidney transplantation reflected the creatinine reduction rate on day 7. Despite the presence of mRNA for these biomarkers in exosomes, their levels did not reflect or predict urinary biomarker levels or the creatinine reduction rate. This is most likely due to the fact that mRNA packaging in exosomes is selective and not always representative of mRNA in the parent cells responsible for biomarker production [131]. In another study, the expression of NGAL in uEVs was higher than in urinary cells and correlated with delayed graft function (DGF) [132]. Urinary CD133^+^ EVs appeared to be reduced in kidney transplant patients with slow graft function and vascular damage, implying kidney stem cell compartment damage [133]. In a rat model of ischemia/reperfusion injury (IRI), urinary AQP1 and AQP2-containing EVs were reduced, most likely due to impaired trafficking and expression of these proteins in renal tubule epithelial cells [134], confirming previous findings of decreased abundance of AQP1 in kidney transplant recipients in the immediate postoperative days [135]. Acute diuresis following kidney transplantation may be caused by a decrease in kidney expression of AQP2, the concentration of which can be estimated from the amount released in uEVs [136].

Immunosuppressive drugs are still required in kidney transplant patients. Despite their known nephrotoxicity, calcineurin inhibitors (CNI) have been considered first-line immunosuppressive agents since their discovery. Chronic CNI toxicity can cause kidney fibrosis, which is dangerous for graft survival. Differentially expressed urinary exo-miRs may be useful markers in kidney transplantation to monitor tacrolimus therapy and graft function. Sixteen exo-miRs were found to be differentially expressed, with miR-155-5p upregulated and miR-223-3p and miR-1228-3p downregulated. MiR-155-5p and miR-223-3p expressions were associated with tacrolimus dose (*p* < 0.05), miR-223-3p with serum creatinine (*p* < 0.05), and miR-223-3p and miR-1228-3p with blood leukocytes (*p* < 0.05). Targets for 12 miRNAs have been predicted to be involved in cell proliferation, apoptosis, stress response, PIK3/AKT/mTOR, and transforming growth factor-beta (TGF-β) signaling pathways [137]. Members of the uroplakin and plakin families were found to be significantly upregulated in patients with chronic CNI toxicity group [138]. Monitoring urinary vitronectin has the potential to become a noninvasive biomarker of fibrotic changes in kidney transplant recipients [139].

Acute rejection is critical for graft survival and occurs at a 10% rate within the first year in most transplant centers, despite improved immunosuppressants [140,141]. Regular monitoring of serum creatinine and proteinuria levels are nonspecific biomarkers, and detectable differences generally indicate that an allograft has already established irreversible injury. Although allograft biopsy is considered the gold standard for transplant injury detection, it is invasive and cannot be used as a serial monitoring method [142]. For these reasons, there is a call for the development of novel biomarkers capable of noninvasively diagnosing acute T-cell-mediated rejection in kidney transplant recipients. Among 458 graft biopsies enrolled in a cross-sectional multicenter study, 46 proteins were found to be overexpressed in patients with a stable graft function, while 17 proteins were found to be overexpressed in acute T-cell-mediated rejection patients. Tetraspanin-1 (TSPAN1) and hemopexin concentrations were significantly higher in acute T-cell-mediated rejection patients than in controls (*p* = 0.009 and *p* = 0.046, respectively) [143]. Tetraspanins are involved in a number of fundamental cellular processes, such as cell adhesion and migration, as well as intracellular signaling and trafficking. They also influence and control various immune-related roles [144]. Increased TSPAN1 expression may be linked to acute T-cell-mediated rejection via interactions with integrins [143]. In patients with an acute cellular rejection, integrated kidney exosome analysis revealed a high level of CD3^+^-EVs and achieved a high detection accuracy (91.1%) [145]. Besides, normalized extracellular vesicle-bound DNA (evDNA) yield (*p* = 0.042) and evDNA copy number (*p* = 0.027) differed significantly between patients with normal histology, rejection injury, and nonrejection injury, with the latter having significantly larger uEVs (mean diameter, *p* = 0.045) and more DNA bound per uEV. Donor-derived DNA was detectable in uEV samples from kidney allograft recipients, but the amount varied greatly. Several evDNA characteristics correlated with clinical and histological parameters (*p* = 0.040) in a proof-of-principle study, indicating that the potential of evDNA as a biomarker for kidney allograft injury should be investigated further [146].

Chronic active antibody-mediated rejection is a particular issue in kidney transplantation, accounting for 25% of graft loss. In a cross-sectional multicenter study consisting of 26 kidney transplant recipients with biopsy-proven chronic active antibody-mediated rejection, 57 kidney transplant recipients with long-term graft survival, and 10 rejection-free matched kidney transplant recipients, six proteins (apolipoprotein A1 (APOA1), transthyretin (TTR), polymeric immunoglobulin receptor (PIGR), hemopexin (HPX), inc-α-2-glycoprotein (AZGP1), and ceruloplasmin (CP)) that distinguished chronic active antibody-mediated rejection from long-term graft survival were chosen. AZGP1 was discovered to be a chronic active antibody-mediated rejection-specific proteomic biomarker that could be identified from the rejection-free control group with matching kidney function, transplant duration, and age [147]. Synaptotagmin-17 (SYT17) levels were higher in an exosomal fraction of urine from chronic active antibody-mediated rejection patients compared with three other histology groups (normal, interstitial fibrosis and tubular atrophy, and calcineurin inhibitor toxicity), and this was linked to the activation of the IL-6 amplifier [148].

Investigating the biological machinery associated with BK virus (BKV) infection in kidney transplantation, proteomics analysis of uEVs showed that kinase was the only gene ontology annotation term that included proteins that were not abundant in BKV (with SLK being the most significantly downregulated protein). Nonlinear support vector machine (SVM) learning and partial least squares discriminant analysis (PLS-DA) identified 36 proteins capable of discrimination (including DNASE2, F12, AGT, CTSH, C4A, C7, FABP4, and BPNT1) between kidney transplant recipients with and without BKV. The proteomic profile of kidney transplant recipients with BKV viruria alone was very similar to that of viremia and viruria [149]. A small cross-sectional multicenter study suggested that urine exosomal BKV-miR-B1-5p and BKV-miR-B1-5p/miR-16 could be used as surrogate markers for BKV nephropathy diagnosis. The AUC values for BKV-miR-B1-5p and BKV-miR-B1-5p/miR-16 were 0.989 and 0.985, respectively, in the ROC analysis for the diagnosis of BKVN [150].

## 4. The Use of Urinary Extracellular Vesicles in Children with Kidney Disease

### 4.1. Kidney Hypoplasia

Using nanoparticle tracking analysis and quantitative proteomics, differentially expressed proteins in uEVs were identified in bilateral kidney hypoplasia, which is characterized by a congenitally reduced number of nephrons. This uEV expression signature reflected decreased kidney function in CKD patients due to congenital kidney and urinary tract disease [16]. Distal tubule and collecting duct-specific MUC1 was one of the molecules found to be reduced in uEVs in kidney hypodysplasia. MUC1 serves a variety of functions in both the normal and injured kidney. Mutations in the *MUC1* gene cause tubulointerstitial disease, which leads to kidney failure [151], and abnormal MUC1 signaling activation is linked to the development of CKD [152]. The reduced MUC1 expression in uEVs implies decreased excretion of classical exosomes due to functional nephron loss. In addition, in kidney hypodysplasia, uEVs had higher levels of proximal tubule-specific maltase-glucoamylase (MGAM). Although the validation cohort did not show a statistically significant increase in MGAM in CKD, it is thought to capture a different aspect of uEVs from decreased MUC1 expression and thus improves diagnostic ability when combined with MUC1 [16]. MGAM, an α-glucosidase, acts as an enzyme in the final step of starch digestion, converting linear regions of starch to glucose [153]. Increased expression of MGAM, like other proximal tubule markers, may be associated with compensatory responses in proximal tubular cells [16].

### 4.2. Focal Segmental Glomerulosclerosis

In adolescents and young adults, focal segmental glomerulosclerosis (FSGS) is a common cause of CKD and ESKD. Although the exact cause of primary FSGS is unknown, it is thought to be caused by circulating molecules, which cause podocyte effacement, atrophy/autophagy, and segmental sclerosis. However, the disease process and glomerular cell interaction are not fully understood [154]. The pathologic diagnosis of diffuse podocytopathy with minimal changes (MCD) is important and common in adults and children with nephrotic syndrome. Light microscopy shows minimal changes, but electron microscopy shows extensive injury to glomerular podocytes with diffuse foot process effacement and loss of slit diaphragms in the absence of electron-dense deposits [106]. The result of these changes is massive proteinuria due to glomerular filtration barrier failure, the integrity of which is critically dependent on the specialized junctional slit diaphragm protein complex linking the interdigitating podocyte foot processes [155]. Auto-antibodies that target the fundamental structural slit diaphragm component nephrin were recently discovered in a subset of patients with noncongenital, childhood, and adult-onset MCD [156].

In a study with 20 FSGS patients and 10 healthy controls, the EVs in the FSGS group were significantly larger than those in the healthy control group. EVs from the FSGS group activated the signal transducer and activator of the transcription 3 (STAT3) pathway, which presumably leads to a dose-dependent increase in mesangial cell proliferation. When mesangial cells were challenged with EVs isolated from FSGS patients, proliferating cell nuclear antigen (PCNA), Ki67, and cell proliferation were also significantly increased [10].

Urinary exosomal miR-193a has been evaluated as a potential biomarker for the diagnosis of primary FSGS in children. Urinary exosomal miR-193a levels were significantly higher in children with primary FSGS (*n* = 8) than in children with MCD (*n* = 5). The AUC of the ROC curve for detecting primary FSGS with urinary exosomal miR-193a was 0.85 [157]. MiR-193a target genes (such as *Mcl-1*) are involved in the regulation of cellular apoptosis and autophagy [158]. miR-193a may promote FSGS progression by regulating autophagy in podocytes [159].

Wilms’ tumor 1 (WT-1), a urinary exosomal biomarker with apparent podocyte specificity, is another promising noninvasive biomarker that can detect early progression and treatment-induced regression of podocyte injury in FSGS or steroid-sensitive nephrotic syndrome. In human subjects, urinary exosomal WT-1 concentrations were significantly higher in FSGS patients compared with healthy volunteers or steroid-sensitive nephrotic syndrome patients. Urinary exosomal WT-1 levels were also significantly lower in patients in remission from either FSGS or steroid-sensitive nephrotic syndrome, or after steroid treatment in six steroid-sensitive nephrotic syndrome patients [160]. Although the potential of urinary WT-1 as a noninvasive biomarker for podocyte injury was demonstrated in active childhood nephrotic syndrome, there was no difference in exosomal WT-1 between FSGS and non-FSGS [161]. WT-1 could be used as an alternative biomarker or to supplement proteinuria/albuminuria. It has the potential to detect podocyte injury, monitor progression, and predict response to therapy, thereby improving care for FSGS and steroid-sensitive nephrotic syndrome patients [160].

### 4.3. Glomerulonephritis

The potential role of CD133 as a urinary biomarker modulated during kidney damage was investigated in both acute and chronic glomerular diseases. During the acute phase of kidney damage, the concentration of CD133 uEVs was significantly reduced in pediatric patients with acute glomerulonephritis, but it was restored after recovery. Patients with chronic glomerulonephritis experienced a similar decrease. ROC curve analysis showed that CD133 uEV values could distinguish between health and glomerular disease. The presence of CD133^+^ uEVs may be a simple marker of normal kidney physiology, providing information on the “reservoir” of regenerating cells within tubules [162].

## 5. Conclusions

The discovery of uEVs has opened up a new field of biomarker research because urine is a noninvasive and readily available biofluid. Their potential application as diagnostic, prognostic, or therapeutic biomarkers for a variety of kidney diseases is currently being investigated. However, some challenges remain. These difficulties include the need to standardize isolation methods, normalize samples, and validate candidate biomarkers [163,164]. In general, interpreting the biological significance of elevated miRNA levels in uEVs is difficult. The miRNA profile found in uEVs may reflect the conditions in the cells of the tissue from which they originated. Some of the miRNAs found in uEVs may be merely waste, while others may be involved in intercellular signaling [91,92]. Whereas creatinine is used to normalize albumin excretion, the use of exosomal biomarkers may be improved by normalizing for an exosome excretion biomarker [160]. When comparing uEV biomarkers between individuals, a measure of nephron mass or uEV excretion rate should be included [25]. The influence of age, gender, diet, comorbidities, proteinuria, and hematuria on uEVs should be further investigated [165]. It is also preferable to develop a high-throughput platform for isolating and analyzing uEVs, such as an enzyme-linked immunosorbent assay [163]. A single urinary miR, like serum biomarkers, is unlikely to have enough diagnostic value to be clinically useful. Multimarker panels will need to be designed and validated in larger studies before they can be used in clinical trials [78]. The findings of the studies discussed in this review must be replicated and extended to larger study groups with longitudinal follow-up because they are frequently derived from a small number of patients. Future research will expand our understanding of the role of EVs in disease processes, but also as novel therapeutics, and as targets for kidney disease treatment [166].

## Data Availability

Not applicable.
